# Acknowledgment to Reviewers of *Toxics* in 2021

**DOI:** 10.3390/toxics10020062

**Published:** 2022-01-30

**Authors:** Toxics Editorial Office

**Affiliations:** MDPI AG, St. Alban-Anlage 66, 4052 Basel, Switzerland

Rigorous peer-reviews are the basis of high-quality academic publishing. Thanks to the great efforts of our reviewers, *Toxics* was able to maintain its standards for the high quality of its published papers. Thanks to the contribution of our reviewers, in 2021, the median time to first decision was 17 days and the median time to publication was 40 days. The editors would like to extend their gratitude and recognition to the following reviewers for their precious time and dedication, regardless of whether the papers they reviewed were finally published:
A. M. TaiwoKirsten C. SadlerAbdolrahman KhezriKi-Tae KimAbigail L. WhitehouseKlára A. MocováAdam M. KieferKlaudia KosekAdelina GamaKoichi UemuraAditya Rio PrabowoKoji ArizonoAgnese GiacominoKongtae RaAgnieszka NowakKoni GrobAgnieszka ParzychKonrad A. SzychowskiAgnieszka SynowiecKostas KasiotisAgnieszka WnukKouji MiyazakiAhmed AbdeenKoutavarapu RavindranadhAida PetcaKristen P. MorieAirton C. MartinsKristin EcclesAkihisa HataKristina Pogrmic‐MajkicAlan DucatmanKrystyna PawlakAlberto MantovaniKrzysztof RóżyłoAleksandra FucicKsenia PazdroAleksandra SzydlowskaKsenija DurgoAleksandra Ukalska-JarugaKunihiko NakaiAleš ObrezaKyoung-Sik MoonAlessandro GranitoKyung Taek RimAlessandro MarroneLadislav MenšíkAlessio MasiLaís F. BerroAlex G. StewartLamiaa M. A. AliAlexander M. ZakharenkoLars D. HylanderAlexander NovikovLászló AntalAlexander ParshinLaura BulgariuAlexander S. LukatkinLaura LoriniAlexandra EstherLaura NarcisoAlexandra HoaghiaLaura Van WinkleAlexandra PehlkenLaurence Lebaron-JacobsAlfredo G. CasanovaLawrence DuffyAli ZaiterLawrence SchellAlica PizentLeif AbrellAlice LuddiLenka DemkováAlicia BoltLeslaw TeperAlicia Gomis-BerenguerLevent BatAliki-Eleni FarmakiLevonas ManusadzianasAlison R. GerkenLianguo ChenAlisson BorgesLiao Hsuan-chiehAlmudena Veiga-LopezLibing YunÁlvaro OsornioLidia DąbrowskaAmanda BuergerLihua ChenAmeer Fawad ZahoorLilah GlazerAna BorotaLin YeAna GorostidiLinda LoretzAna LeahuLing YuAna Virginia FilgueirasLisa A. MurphyAnca DinischiotuLiudmyla SoldatkinaAndrás PappLixin YangAndré EsserLluis Luján LermaAndre Montiero Da RochaLoka PenkeAndrea Adamcakova-DoddLorena CorettiAndrea AnnibalLorena PerićAndrea Augusto SfrisoLoris PietrelliAndrea C. GoreLouis Kuoping ChaoAndrea CeciLouis TremblayAndrea GersonLuca CarenaAndrea Roxanne J. AnasLuca FinettiAndrea SevcovicovaLucia RoccoAndrea SfrisoLuciana PereiraAndrea SpinazzèLucie JašíkováAndrea VanniniLudek BlahaAndreia FreitasLudmila V. PuchkovaAndrés Posso-TerranovaLuigi RiganoAndrés Rodríguez-SeijoLuigi VimercatiAndrew ChappellLuis Andre MendesAndrew PoveyLuis D. BoadaAndrzej HutorowiczLuís MartinsAneta Ostróżka-CieślikLukáš RichteraÁngel J. GutiérrezŁukasz ChrzanowskiAngel R. BarchukLuke MontroseAngela StufanoLuminita CrisanAngelo MorettoLutz WeihermüllerAngelo SpenaLynne A. FieberAngiola ForleoM. Carmen Martínez-CuestaAniello FrancescoMagdalena PizońAnja StajnkoMagdi El FerganiAnna ForsbyMagnus EngwallAnna Maria NuzzoMahmoud El-MaghrabeyAnna MuratovaMaja ŠrutAnna ParusMałgorzata SzostekAnna PilutinMałgorzata TeleckaAnna Szafranek-NakoniecznaMałgorzata WolskaAnna TurekManfred NeubergerAnnalisa AbballeManhai KavvalakisAnnalisa Di BernardinoManuel Joaquim Sabença FelicianoAnnegret Biegel-EnglerManuel MatosAnnelise J. BlombergMarc EkkerAnnemarie StroustrupMarcelino Miguel OliveiraAntonella GuzzonMarcello VeigaAntonia Concetta EliaMarcin W WochAntonietta GattiMarco MaielloAntonina ArgoMarco PelinAntónio CuradoMarco RomanAntonio Peña-FernándezMarcos A. L. Dos ReisAntreas AfantitisMarek WiergowskiApolonia SieprawskaMaren Ortiz-ZarragoitiaAránzazu SánchezMargareta TorqvistAriela BurgMargot ErnstAristeidis TsagkarisMaria Alejandra DecimaArkadiusz NędzarekMaria AstolfiAshaf Aly HassanMaría Del Pilar SuárezAshutosh PandeyMaria Elena FerreroAthanasios KungolosMaria Helena FernandesAthina AngelopoulouMaria HoluszkoAttila ZsarnovszkyMaria J. Casanueva-MarencoAurélie GoutteMaría Jesús HerreroAustin ScircleMaria L. PerepechaevaBarbara FutaMaria Lígia SousaBartosz WielgomasMaria Morel-EspinosaBashar AmerMaria RicciardiBaubak BajoghliMaría Teresa Barral SilvaBeatrice CampanellaMaria UrbańskaBeatriz FerrerMarianne KollerBeatriz Sevilla-MoránMarie-Cecile G. ChalbotBecky TalynMarija StjepanovićBenjamin C. BlountMarina BurachevskayaBenoit NemeryMarina J. Orlova-BienkowskajaBernardina ScafuriMarinela IntaBernd GöckenerMario FernándezBernhard RyffelMario Vincenzo RussoBhagavatula MoorthyMarios G. KostakisBianca UngerMarios SpanakisBłażej KudłakMarisa NicolaiBo DongMarissa SobolewskiBogdan Gabriel ȘlencuMark CherrieBoris GuyotMark ClemensBożena BukowskaMark J. WilsonBranka LevajMark S. CastroBrian BirchMarko GerićBrian De La FranierMarkus W. SigristBrittan ScalesMarlena RobakowskaBrunislav MatasovićMarta D’AmoraBuckley McCallMarta LombóByumseok KohMarta MarmiroliCai ZongMarta OliveiraCarla ViegasMarta OteroCarlo PorfidoMarta Pérez GonzálezCarlos BarataMartial CaillaudCarmelia Mariana Dragomir-BălănicăMartin DanaherCarmen HerenciaMartin KharraziCarmen NicolaeMary N. SheppardCarmine ApollaroMasato HondaCasimiro S. MunitaMasayuki TakigawaCatarina SeabraMassimo IorizzoCecilia BossaMasumi SuzuiCélia VenturaMateusz JankowskiChang GuoMateusz S. WietechaChang HeMathieu Di MiceliChanggu LeeMathieu GautierChanghyun RohMatilde Maria Moreira Dos SantosChanglun TongMatteo FabbriChaoqi ChenMatteo PaciniChen Hsin ChangMatthaios P. Kadela-TomanekChen-Chi TsaiMatthias KochCheng-An TaoMaurizio GualtieriChiara CavaliereMaurizio ManeraChiara Maria MottaMaurizio MasciChiara RussoMayukh BanerjeeChien-Sen LiaoMd. Shiblur RahamanChristina EmmanouilMd Tabish NooriChristine CurranMelanie BertinChung-Der HsiaoMelinda KrebszChunyong ZhangMelissa Anne SuterClara Amalie Gade TimmermannMelissa FariaClara IannuzziMerv FingasClaudia Campillo-CoraMichael P. SchmidtCláudio ParenteMichael PoteserConcetta PirontiMichaela ZeinerConstantin ApetreiMichał GąsiorekConstantin YanicostasMichal NiemczakCooke MarcusMichał RejdakCorina PaulMichał WichlińskiCostantino CiallellaMichel YeglesCostanza MajoraniMichelle HladikCostanza RovidaMiguel A. OrtegaCristian Ferreiro SantisoMiguel Ángel Sogorb SánchezCristiana RadulescuMiguel MouratoCristiano CasucciMikhail GantsevichCristina AlmeidaMikko HerralaCristina NicaMilan BrankovCristoforo PomaraMinfang ZhangČrtomir PodlipnikMin-Kyeong YeoCynthia RiderMin-Suk KimDaniel DuneaMiran BrvarDaniel Garcia-SoutoMirosław AndrusiewiczDaniel P. PotaczekMirosław KarpińskiDaniel Sobrido-CameánMirta MilicDaniel VollprechtMohamed AshryDaniela ValensinMohd Zuwairi SaimanDaniela ZuzoloMokgaotsa Jonas MochaneDaniela-Saveta PopaMonia PeruginiDaniele Del BuonoMonika JakubusDanielle CarlinMonika Kadela-TomanekDanielle PhilibertMontserrat SoléDanijela Đukić-ĆosićMorteza Hasanzadeh KafshgariDanijela PetrovicMuhammad NadeemDapeng PengMuhammad UsmanDaqiang YinMuriel VayssadeDario AchaMustafa Al AukidyDave BoucherMyung-Bok WieDavid A. JettNadja FreundDavid DoraNałȩcz-Jawecki GrzegorzDavid E. JacobsNancy HopfDavid Fernández-CalviñoNanna B. HartmannDavid G. CalatayudNatalia El-MerhieDavid Lozano-PaniaguaNatalia GrubaDavid M. CrizerNathan LothropDavid Q. AndrewsNaudia GrayDavid W. InouyeNavatha AlugubellyDavide CicalaNeha MehtaDavide VignatiNellie TsipouraDavor ŽelježićNiamh QuinnDawei ShiNicholas HerkertDecai JinNicola MontemurroDiana LupuNicolae GicaDiego BaragañoNicolás MontalbettiDiego BaronioNicoletta LotrecchianoDiego IaconoNicusor-Flavius SimaDimitra KaraliNika LovšinDimitris KaskaoutisNikita MoiseevDiptiman BoseNikolas AntonatosDirk LachenmeierNikolay SolovyevDivyapriya GovindarajNikolett UzingerDmitry VlasovNishimoto YukoDomenico GadaletaNoriatsu OzakiDomenico SurianoNoritaka KatataniDominik PoradowskiOana CadarDominika DabrowskaOihane Diaz De CerioDominika ŚwięchOleg Yu. SheshukovDominika ZającOlga TrhlíkováDon GreenOlivier SorgDonggang XuOmid AkhavanDóra ParóczaiOndrej CehlárDorota Olszewska-SłoninaOrestis SchinasDragos HorvathOrthodoxia MastrogianniDuc-Hiep NguyenOsvaldo ZariviDusan JandackaOthmane MerahEbrahim LariPablo Antonio Lara MartínEdit HermeszPablo TejedoEdward HenselPadmini S. J. KhedoeEdwin Westreicher-KristenPanagiotis BerillisEfi LevizouPankaj ChowdhuryEfstratios Stratos NikolaivitsPatrícia PereiraElena BonciuPatrick LegembreElena GregorisPatrizia BovolinElena PorzioPaul M. StemmerElena ViganòPaul T. J. ScheepersEleonora MazzuccoPaula Elisa Redondo HasselerharmElijah PetersenPaule VasseurElisa BustaffaPaulina BednarczykElisabeth RiegelPaulina RusanowskaElisabetta BertolPawel PohlElisabetta CarataPaweł SiudemElizabeth MullinPeter FranklinEls Van HoeckPeter MaskellElżbieta Gumienna-KonteckaPeter ParsonsEmanuel BaltagPetru NegreaEmese GalPhilippe GuerreEmilia NeagPhillip L. WilliamsEmmanuel Obeng-GyasiPietro FerraraEnrica AllevatoPiotr A. GaudenErica L. MajumderPiotr KaminskiEster PapaPrabha RanasingheEuan R BrownPrabhakar SharmaEui-Bae JeungPraveen KumarEun Hea JhoPura Marín SanleandroEunsook LeeQuoc Cuong DoEva PertileRadek ŽouželkaEva SingovszkaRafael Prado-GotorEvangelos KarvelasRaffaello PapadakisEwa OleńskaRaj PaudelEwelina CzubackaRajendra PrasadEwelina PatyraRamon LavadoFabian FischerRamona ZgavarogeaFabiana CoramiRanka GodecFabio VaianoRavi VattepuFaraz AhmadRaymond PalmerFarhan R. KhanRazieh SadraeiFarzana SabirReinhard OertelFatima JesusRemigiusz BąchorFederico Maria RubinoRhianna K. MorganFernanda OduberRicardo MarcosFernanda VilarinhoRichard D. FoustFilbert TotsinganRichard Steven PappasFilippo CiabrelliRichard Steven PappasFiras KobeissyRick LangleyFlorian GambusRita Carina BichoFlorin-Constantin MihaiRita SousaFobang LiuRizwan AnsariFotis IliopoulosRobert H. RiceFrabrizio OlivitoRobert TureskyFrancesca CoppolaRoberta D’AurelioFrancesca GoriniRoberta RussoFrancesco EspositoRoberto BaroneFrancesco OrienteRoberto Della PergolaFrancesco TriggianoRoberto MartinsFrancisco SolanoRoberto PaciottiFranco MutinelliRoberto Romero-GonzálezFrank WelleRobyn L. PrueittFrederick WilliamsRoger ChongFumiki TakahashiRona KarahodaGabi DrochioiuRosa SinisiGabriel PlavanRosita GabbianelliGagandeep KaurRostislav StreletskiiGanna PetrukRoxana Racoviceanu (Babuta)Gary L. FrancisRuairi MacNamaraGemma VillorbinaRui DingGeorgia KastrinakiRui SongGergő KallóRwik SenGiacomo Carlo SturnioloSabina PodlewskaGianfranco FrigerioSabina ZiembowiczGianfranco SantovitoSalvatore BarrecaGilberto BindaSamir BarghoutGilles-Eric SeraliniSamreen SiddiquiGiovanna CalabreseSamuel CaitoGiovanni LanteriSamuel NutileGiovanni RollaSanaz OrandiGiovanni VintiSang-Sup LeeGiuliana SolinasSara BernardiGiuseppe LungarellaSarah F. EvansGiuseppe ManciniSascha K. ManierGiuseppe PanarelloSaumik Saumik PanjaGiusy TassoneSergei ChernyakGloria IsaniSergey ZhironkinGo SuzukiSergio MinucciGongbo ChenSeunghwan YangGopal BeraShaofei JiGopinath MuruganandamSharanya KalasekarGoran MitulovicShegufa ShetranjiwallaGouse Peera ShaikShenghong YangGovindan MalarvannanShigeki MatsubaraGrażyna JanikowskaShigeo MuroGrażyna KowalskaShih-Yi HuangGregorio GarciaShogo OzawaGrzegorz SchroederShu-Qin YuGrzegorz TylkoSibylle ErmlerGuangce WangSigrid Kusch-BrandtGuangxu LiuSilvana FioritoGuido PanzarasaSilvano DragonieriGunnar BoysenSilvia LacorteGunnar ToftSilvya Stuchi Maria-EnglerGuohui SunSimona CandianiGuotao PengSimona IodiceGurusankar SaravanabhavanSimona RendaGwenaël RolinSimone CantamessaHa Ryong KimSimone MarzedduHaishu LinSipi SeiskoHaiyuan ZhangSiyuan WangHaniyeh JalalipourSławomir SułowiczHanna MojskaSnježana Herceg RomanićHann-Chorng KuoSofiane BoudaliaHanns MoshammerSónia GonçalvesHaoran ZhaoSonia SentellasHarada KoujiSophia BarinovaHarald G. DillSophie NdawHasintha WijesekaraSoraya Paz MontelongoHatzianestis IoannisSotiris I. PatsiosHelen CrabbeSreeja SarasammaHelmut GreimSreenivasa C. RamaiahgariHengyi XuStacey E. AndersonHenryk KozłowskiStefania PaduanoHenryk MazurekStefanie Hessel-PrasHerbert Ryan MariniStephanie PadillaHideko SoneSteven O’HaraHiroe Hara-YamamuraSubhabrata DasHiroki TeraokaSubham DasguptaHitoshi AsanoSudhir KotnalaHolger LutzeSudip MukherjeeHolly N. WilkinsonSudip PaulHongbing WangSung-Min KangHouda BerradaSunit SinglaHow-Ran GuoSusana SantosHrvoje PavlovićSven WürtzHubertus P. A. JersmannSvetlana N. SushkovaHugo A. L. FilipeSvetlana SemenovaHugo Saldarriaga-NoreñaSylwester SommerHui WangSylwia BudzynskaHui-Ju LinTadeusz SiwiecHung Quang TranTaehyun RohHung-Yi ChuangTaisen IguchiIan R. FalconerTaisuke TomonagaIgnacio Moreno-GarridoTakafumi SetoIgnacy KitowskiTakashi NomizoIgnasi SanahujaTakashi SatoIlir ShehuTakehiro MichikawaImran AliTakuomi HosakaIna RissanenTallent DadiInga ZinicovscaiaTamara JakovljevićInge WernerTamara WrightIngrid FalnogaTatiana KaletovaIn-Sul HwangTatyana KrupnovaIoana SurTatyana MinnikovaIoannis MessinisTeresa HortonIrena Baranowska-BosiackaTerry TetleyIrena Brčić KaračonjiTeuta MuratiIrene CampiThais PosserIrene Gomes Câmara CamachoTheodoros GlytsosIrene Torres-SánchezTheoni MargaritopoulouIrina BlinovaThomas BøhnIrina LyanguzovaThomas KniggeIsabel LopesThomas NiglIsabel María MorenoThomas SchmidtIsabella ZanellaTiago SimõesIulia ChiciudeanTiina TitmaIulian PojarTimur Yu. MagarlamovIva SovadinovaTina KeglIvan Gláucio Paulino-LimaTin-Tin Win-ShweIvan P. PozdnyakovTobias StraubIvana MirkovTodd LeffIvanka TenevaTomas ErbanIvo DostálTomas PospisilIvona NuićTomáš VyhnánekIwona ZawiejaTomasz KakarekoIyyakkannu SivanesanTommaso FilippiniIzabela Szymczak-PajorTomohiko IsobeJacek AntonkiewiczToshihiko HanaiJack RogersTsung-Yun LiuJackie WrightTzu-Hsuen YuanJake MartinUdi VazanaJakob WolframUrszula WydroJakub CiazelaVaida ŠerevičienėJames DevillersValentina CarozziJames MatthewsValentina Christova-BagdassarianJames O’CallaghanValentina GalbiatiJan KubesValeria GasperiJan SchinkötheValéria Guimarães Silvestre RodriguesJana Faganeli PucerValéria Stefania Lopes-CaitarJane MunckeValeria TirantiJanez MravljakVan Geer FransJanka BábelováVanesa Santás-MiguelJanna G. KoppeVanessa BrinkmannJaroslav MrázVasco BrancoJason MagnusonVeronika AleksandrovychJason O’BrienVeronika GrauJason RaineVeronika KhairullinaJavier González-SálamoVeronika Pedrini-MarthaJavier Perez-GarciaVesna Cerkvenik-FlajsJean-Baptiste RenardVesna PeršićJeanette HowardVesna RastijaJennifer StowellVictor Bocos-BintintanJess W. EverettVíctor M. LeónJesús Sánchez-MárquezVijay BoggaramJi Hoon SeoViktoriya S. SidorenkoJian FuVinay ShuklaJianfang WangVincent MarkowskiJiangang ChenVincenzo CavalieriJiayuan ZhaoVincenzo MicaleJieun LeeVineeta TanwarJill A. JenkinsVinod KumarJingshu GuoVinoth K. SittaramaneJin-Yong LeeVivek DharwalJith K. ThomasVladimir Gus’kovJoachim MutterVladimir PitschmannJoana F. MonteiroWalter Christopher WilfongJoana PrataWalter G. BradleyJoanna Gdula-ArgasińskaWalton SumnerJoanna KisalaWataru MiyazakiJoão De Sousa ValenteWayne HallJoel PereiraWei-Chun ChouJohannes BallauffWen-Der WangJohannes LützenkirchenWen-Yinn LinJohn E. FrenchWiktor HaleckiJolanta B. ZawilskaWilliam M. GwinnJolanta FliegerWinfried NeuhausJonathan OkonkwoWing-Kee LeeJong-Chin HuangWladyslaw JanusJongwoon KimWojciech KieratJörg UhlemannWojciech LeppertJorge Verdú-AndrésWojciech PiekoszewskiJosé Antonio RodríguezWolf-Dieter MuellerJosé Herrera-MeliánXiang LiJosé Luis Rodríguez GutiérrezXiaofeng WenJose Maria SantosXiaojun LuoJosé Simão Antunes Do CarmoXiaotian TangJoshua EdwardsXiaozhe YangJosip BarišićXin CuiJoyanto RouthXue BaiJoyce TsujiXuexiang HeJudit Oliver-MeseguerYang ZhouJudith E. McDowellYaodong HeJulia D. FineYaser Hosny ElewaJulia M. MalyshYayoi KobayashiJulie PellerYiin Lih-MingJulien LegrosYlenia MieleJuliette MartinYng-Tay ChenJun UdagawaYogesh SainiJung-Ren ChenYohannes YihdegoJungsu ParkYoko SuzumotoJustin AlbertYong LongJustyna Płotka-WasylkaYoshiharu FujiiKai ZhangYoshika SekineKang WangYoshiteru MorioKanno YuichiroYounglim KhoKarel HrichYu Ait BamaiKarl PayneYu ZhangKarla Oyuky Juarez-MorenoYuguang ZhouKarol SikoraYuling XieKarol SteckiewiczYunru JuKarolina Matej-ŁukowiczYun-Shan LiKatarzyna AffekYves CherelKatarzyna Marciniak-ŁukasiakYves CombarnousKatarzyna PentośYvonne SunKatarzyna SutkowskaZaneta PolkowskaKate F. JohnsonZbigniew KobusKaterina GiouriZeljka LoncaricKazuomi SatoZhaowei ZhangKazushi NoroZhihao WuKehinde BabatundeZhixiang ZhangKehinde ErinleZhiyuan FengKellie A. FayZhongxiu SunKentaro MisakiZhongzhi ChenKenza MamouniZhuang WangKeri BarksdaleZhuoran MaKevin WelchZiarno MałgorzataKeyvin DarneyZunwei ChenKhaled Mohammed GebaZunyao WangKhanneh Wadinga FombaZuzanna BielanKimberly BergerZuzanna RzepkaKinga GawelZygmunt KowalskiKirsi H. Vähäkangas



